# Label-free biosensing with singular-phase-enhanced lateral position shift based on atomically thin plasmonic nanomaterials

**DOI:** 10.1038/s41377-023-01345-6

**Published:** 2024-01-01

**Authors:** Shaodi Zhu, Rodolphe Jaffiol, Aurelian Crunteanu, Cyrille Vézy, Sik-To Chan, Wu Yuan, Ho-Pui Ho, Shuwen Zeng

**Affiliations:** 1 Light, Nanomaterials & Nanotechnologies (L2n), CNRS-EMR 7004, University of Technology of Troyes, 10000 Troyes, France; 2 Department of Biomedical Engineering, The Chinese University of Hong Kong, Shatin, New Territories, Hong Kong, China; 3 XLIM Research Institute, UMR 7252 CNRS/University of Limoges, 123, Avenue Albert Thomas, Limoges, France

**Keywords:** Nanophotonics and plasmonics, Optical sensors

## Abstract

Rapid plasmonic biosensing has attracted wide attention in early disease diagnosis and molecular biology research. However, it was still challenging for conventional angle-interrogating plasmonic sensors to obtain higher sensitivity without secondary amplifying labels such as plasmonic nanoparticles. To address this issue, we developed a plasmonic biosensor based on the enhanced lateral position shift by phase singularity. Such singularity presents as a sudden phase retardation at the dark point of reflection from resonating plasmonic substrate, leading to a giant position shift on reflected beam. Herein, for the first time, the atomically thin layer of Ge_2_Sb_2_Te_5_ (GST) on silver nanofilm was demonstrated as a novel phase-response-enhancing plasmonic material. The GST layer was not only precisely engineered to singularize phase change but also served as a protective layer for active silver nanofilm. This new configuration has achieved a record-breaking largest position shift of 439.3 μm measured in calibration experiments with an ultra-high sensitivity of 1.72 × 10^8^ nm RIU^−1^ (refractive index unit). The detection limit was determined to be 6.97 × 10^−7^ RIU with a 0.12 μm position resolution. Besides, a large figure of merit (FOM) of 4.54 × 10^11^ μm (RIU∙°)^−1^ was evaluated for such position shift interrogation, enabling the labelfree detection of trace amounts of biomolecules. In targeted biosensing experiments, the optimized sensor has successfully detected small cytokine biomarkers (TNF-α and IL-6) with the lowest concentration of 1 × 10^−16^ M. These two molecules are the key proinflammatory cancer markers in clinical diagnosis, which cannot be directly screened by current clinical techniques. To further validate the selectivity of our sensing systems, we also measured the affinity of integrin binding to arginylglycylaspartic acid (RGD) peptide (a key protein interaction in cell adhesion) with different Mn^2+^ ion concentrations, ranging from 1 nM to 1 mM.

## Abstract (web summary)

We developed a novel label-free plasmonic biosensing configuration for sub-femtomolar (10^−16^ M) small molecule detection with an ultra-high sensitivity of 1.72 × 10^8^ nm RIU^−1^ and a large figure of merit (FOM).

## Graphical abstract

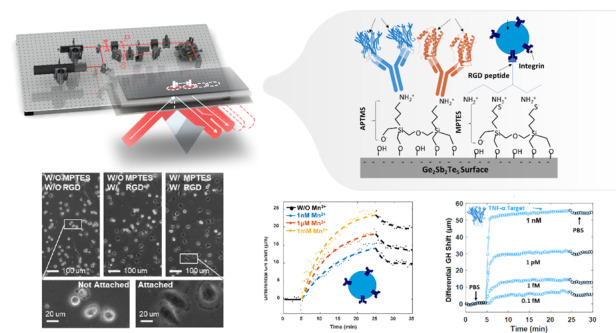

## Introduction

As the leading cause of disease-related fatalities^1^, cancer has been extensively studied for decades to find a more effective diagnostic approach. Assays that detect cancer markers, such as enzyme-linked immunosorbent assay (ELISA), are commonly employed to obtain the phenotypic information in cancer diagnosis^2,3^. Nevertheless, the detection of cancer markers at early stages remains a challenge for conventional assays due to the extremely low concentrations in body, which range from femtomolar (fM) to picomolar (pM)^4–6^. Therefore, the development of techniques with higher sensitivity is necessary for effective cancer screening. In addition to the clinical practices, the biomolecule detection is also essential in basic biology research. One example is the evaluation of molecule binding kinetics, which is critical for developing new drugs and understanding the behavior of biological systems^7–9^. Since the kinetic analysis has to minimize the interference from labeling, such evaluation not only requires a sensitive molecule detection but also a direct measurement without additional contrast/amplifying labels.

Featured by high sensitivity, capability of real-time, label-free detection, and immunity to electromagnetic interference, plasmonic sensors based on surface plasmon resonance (SPR) effect have been widely used in biosensing^10,11^. Conventional plasmonic sensors based on Kretschmann configuration interrogate the change of resonant angle or wavelength where the light has the maximum absorption^12^. The angle/wavelength at which the resonance occurs is dependent on the refractive index of the analyte on metal-dielectric interface. The sensing depth of SPR sensors is typically within hundreds of nanometers, making them particularly suitable for sensing the reaction on surface such as the binding of biomolecules^10,13^. The reported biomolecule detection limit of conventional angle-/wavelength- interrogating SPR instruments are in the range from ng to pg mL^−1^, approximately at picomolar (pM) level^14–16^. However, most of the ultrasensitive detection demonstrated through these instruments requires secondary amplifying labels such as gold nanoparticles, which complicates the test procedure and restricts the application scenarios.

Since the optical phase of reflection undergoes an abrupt drift at the resonance angle, interrogating the reflection phase at resonance is more sensitive than measuring the reflection intensity. The recent phase-interrogating setups based on interferometry have demonstrated one order better detection limit^17,18^. Meanwhile, studies on Goos-Hänchen (GH) shift have paved a new way for phase-sensitive biosensing, which refers to a lateral position shift of the light beam after the total internal reflection^19^. This shift is caused by the interference of the wavefronts that make up the beam. The measurement of GH shift can be obtained with beam position detector such as quadrant sensor or charge-coupled devices (CCD)^20–22^. This indirect phase interrogation method not only simplifies the sensing setup than interferometry, but also provides a potentially higher sensitivity as it measures the higher order form of phase. The GH shift distance is proportional to the differential of reflection phase change to incident angle ($\partial \Phi /\partial \theta$, see Eq. 1), which can be evaluated according to the theory proposed by Artmann et al.^23,24^. However, the GH shift measured in a typical total internal reflection (TIR) interface was only in the range of 5–10 μm^24^. Therefore, to obtain a significant GH sensing contrast, the optical phase changing rate on sensing substrate requires a much faster evolution.

Recent studies on topological phase singularity showed a novel approach to enhance phase change^25–28^. The phase of reflection can be treated as the angle of complex reflection coefficient according to Snell’s law. Due to the topological nature of phase on reflection coefficient plane, plasmonic substrate’s phase drifting will be sharpened in approaching the darkness point of resonance. Subsequently, a steep ***π*** jump in phase, so called topological phase singularity, can be obtained by enhancing the light absorption at the darkness point.

In this work, we obtained a further enhancement of the phase singularity on plasmonic sensor for GH sensing. Two rules were summarized for sensing substrate design according to the relationship between SPR reflectance curve, phase curve, and GH sensitivity. Firstly, the suppressed reflection will accelerate the phase change as discussed above. Thus, a giant GH shift is expected at the quasi-zero-reflection point of resonance spectrum where the phase-changing rate is maximized. Secondly, narrowing the SPR curve with a higher quality factor also contributes to the enlarged phase changing rate and GH shift, since it accelerates the reflectance change near the minimal reflection point.

For practical implementation, an atomically 1-nm thin layer of amorphous Ge_2_Sb_2_Te_5_ (GST) was precisely engineered as the capping material on silver nanofilm. The absorption of this designed GST layer in visible to near-infrared (NIR) range allows a lower reflectance at resonance than bare metallic substrates, which eventually results in the topological darkness. Besides, the 1-nm thin GST capping was demonstrated for the first time to be significantly effective in protecting the underneath metal from degeneration. Despite the lower stability in aqueous solutions for bare silver, it offers a better sensing performance than commonly used gold substrates such as narrower SPR dip and higher angular sensitivity^29^. The validation of this capping layer allows the practical application of silver-based substrate and more flexible choice of active plasmonic materials to further improve the sensing ability.

This novel hybrid silver-GST plasmonic substrate has led to an advanced GH sensing performance in terms of both sensitivity and the detection capability of trace level biomolecules. In calibration experiments using glycerin solutions, the experimental sensitivity of our silver-GST sensing substrate at 785 nm was 1.72 × 10^8^ nm RIU^−1^ and the maximal GH shift was 439.3 μm. The linear range of the sensor response was 1.2 × 10^−3^ RIU (1% w/w of glycerin). In the targeting experiments of cancer markers that functionalized with, we demonstrated the detection of two cytokines, i.e., Tumor necrosis factor alpha (TNF-α, 17.3 kDa) and Interleukin 6 (IL-6, 20.9 kDa), at the concentration of 0.l fM. The corresponding molecule density on sensing region (10 × 10 mm) was evaluated at 0.2 zeptomole per mm^2^ within a 200-μL flow chamber. The binding kinetic between a transmembrane protein, integrin, and its corresponding ligand, Arginylglycylaspartic acid, was investigated as well. The results verified the affinity enhancement of integrins with increasing concentration of Mn^2+^ ions in real-time and executed automatically. These experiments validated our sensing platform as a versatile tool for both clinical and research scenarios.

## Results

### Experimental measurement of lateral position shift

**Fig. 1:**
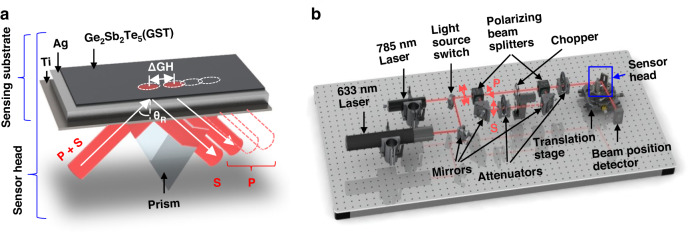
Principle and optical setup of singular phase enhanced plasmonic sensor. **a** Schematic of the sensor head using Silver-GST sensing substrate. The incident light through a prism was coupled to the sensing substrate for surface plasmon excitation. The lateral position shift difference between p- and s- polarized light is denoted as ΔGH. At resonance angle θ_R_, the reflected p-polarized light undergoes a giant lateral position shift that is sensitive to refractive index change. **b** The setup for measuring the lateral position of p- and s-polarized light that reflected from the sensor head. The wavelength of incident light is switchable between 785 nm and 633 nm

Different from conventional angular-/intensity- interrogating methods, this work utilizes the lateral position shift to sense the refractive index change. Such lateral position shift can be observed on a linear-polarized incident beam after the total internal reflection, which is known as Goos-Hänchen shift (GH shift). The GH shift distance is derived by the differentiation of the reflection phase change at certain incident angle^23^. Such position shift on plasmonic substrate can be significantly enlarged from the drastic phase change at resonance angle ${\theta }_{R}$. As illustrated in Fig. 1a, the positions of reflected p- and s- polarized light are separated due to their difference in phase. Since only the p-polarized light, as an electrical field oscillation perpendicular to the sensing substrate, can induce the resonance with surface plasmon. The sudden phase retardation introduced by the resonating plasmonic interface only occurs for p-polarized excitation. Therefore, the GH shift on p-polarized light is giant and sensitive to refractive index change while the shift on s-polarized light is neglectable.

In this work, such phenomenon was utilized for sensing by comparing the position difference between p- and s- polarized light, denoted as differential GH shift ($\Delta {GH}$, see Eq. 1).

1 $$\Delta {GH}=-\frac{{\lambda }_{1}}{2\pi {n}_{1}}\,\left(\frac{\partial {{\rm{\phi }}}_{p}-\partial {{\rm{\phi }}}_{s}}{\partial \theta }\right)$$

Here, ${\lambda }_{1}$ and ${n}_{1}$ refer to the wavelength of incident light and the refractive index of prism; $\theta$ and ${\rm{\phi }}$ are the incident angle and the phase shift of reflection, respectively.

**Fig. 2:**
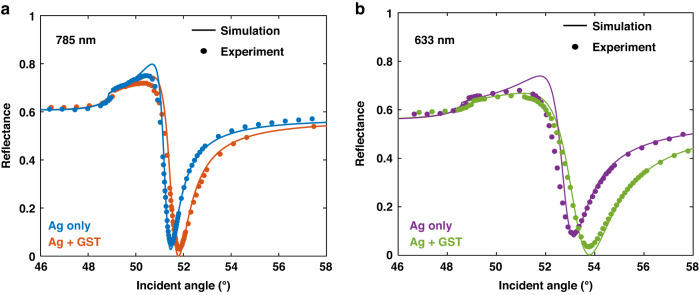
The reflectance calibration of sensing substrate. **a**, **b** The experimental and simulated reflectance curves of silver and silver-GST substrates at 785 nm (**a**) and 633 nm (**b**)

The sensing layers shown in Fig. 1a were coated on sapphire slides using DC magnetron sputtering and coupled with a SF11 prism. The materials from top to bottom are amorphous Ge_2_Sb_2_Te_3_ (GST, 1 nm), Silver (40 nm), and Titanium (7 nm). The thickness was validated by matching the reflectance curve (Fig. 2) and digital holography microscope (see Fig. S1). Besides, a substrate without GST layer was fabricated for comparison. Herein, the titanium was used as adhesion layer to enhance the stability and integrity of materials coated upon. Silver was selected as the main material for generating surface plasmon. Compared to the mainstream gold substrates, silver has a narrower dip in angular SPR spectrum and more abrupt phase change at resonance^30^. The atomically thin GST layer was precisely engineered to maximize the light adsorption and sharpen the phase change at resonance angle which results in a larger GH shift^12,31^. Also, the GST capping can reduce the oxidation of silver that prolongs the lifetime of the sensing substrate. In durability test shown in Fig. S2, the GST-capped silver substrate was demonstrated to resist 24 hours of immersion in buffer without observable reflectance change in visible-NIR range. In contrast, the degeneration of bare silver resulted in a reflectance decay of 60%.

Figure 1b illustrates the overview of the homemade optical setup for measuring the differential GH shift. In this work, we adopted a 633-nm He-Ne laser (HNL100LB, Thorlabs Inc., France) and a 785-nm laser diode (RO-USB-785-PLR-80, Coherent Inc., France) to assess the performance of sensing substrate in visible-NIR range. The incident light was split into p- and s- polarized light and then merged through a pair of polarizing beam splitters (PBS, PBS202, Thorlabs Inc., France). Thus, a chopper (SRS542, Stanford Research Systems Inc., USA) placed between the PBS pair can subsequently switch the light polarization to offer a periodic comparison in lateral beam positions. This is essential for the continuous monitoring of refractive index change. The sensor head was fixed at the resonance angle on a translation stage to obtain the maximal differential GH shift and the highest sensitivity. The position of reflected beam was interrogated by a lateral effect position sensor (PDP90A, Thorlabs Inc., France). The noise characterization given in Fig. S3 indicates that the detector resolution after a low-pass filtering is 0.12 μm, where the filtering period is set to be 5 seconds with real-time raw signal monitoring. Here, all the biosensing data collected by this detector was acquired for every 0.05 s and simultaneously processed by the filtering module.

### Characterization of the sensing substrate

In this work, we first characterized the sensing substrate in the aspects of reflectance curve, phase change, and differential GH shift. The variation of operating wavelength and the existence of GST layer are also investigated to explore the optimization factors of sensing performance. The theoretical model for calculation is introduced in the Methods section.

Figure 2a, b compared the difference in reflectance between the substrates with and without GST layer at both 633 nm and 785 nm. These curves were calculated and experimentally verified with pure water that has a refractive index of 1.3317 at 633 nm and 1.3296 at 785 nm. The sensing substrates operating at 785 nm showed 3-4 orders of magnitude attenuated reflectance at resonance angle and by two times narrower SPR dips (FWHM, full width at half maximum) compared to these at 633 nm. In addition, the deposition of GST layer led to a further deeper SPR curve. With 785 nm excitation, the designed silver-GST substrate can obtain a quasi-zero reflectance at 1.03 × 10^−6^. All performance comparison with details between the substrates has been listed in Table S1.

**Fig. 3:**
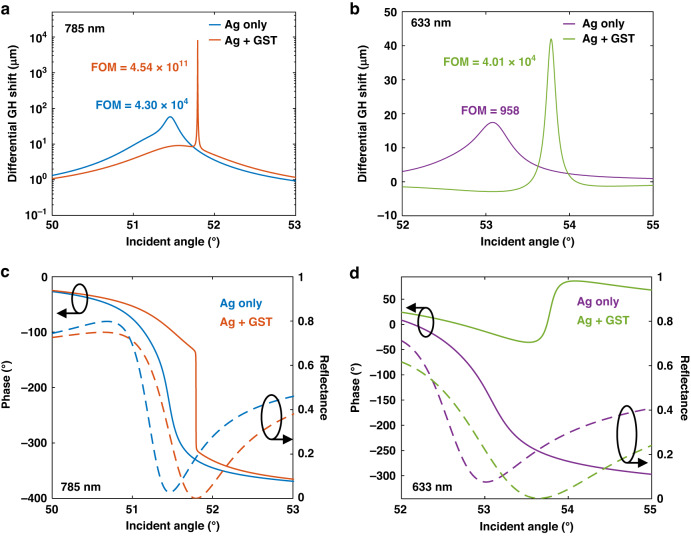
Differential GH shift enhancement reflects the phase singularity from topological darkness. **a**, **b** Comparison of differential GH shifts between silver and silver-GST substrates. The Y axis of (**a**) was plotted in logarithm. The GH shift curve in (**b**) was flipped from negative to positive for better comparison. **c**, **d** The calculated phase change (solid curves) in comparison with reflectance (dash curves) at 785 nm (**c**) and 633 nm (**d**). The calculation is based on the transfer matrix method using parameters listed in Table S2

As shown in Fig. 3a, b, the phase singularity from topological darkness can be intuitively visualized by the differentiation of phase, in the form of GH shift (Eq. 1). A giant peak of differential GH shift from the singular phase jump can be found on silver-GST substrate with 785 nm excitation. The calculated peak GH shift is 7930.4 μm, over 100 times higher than those with silver substrates and silver-GST substrates at 633 nm. Besides, the width (FWHM) of the GH peak on silver-GST substrate is reaching to the level of 0.001°, over 200 times narrower than that with silver substrate alone (0.203°). Also, the maximum GH signal is one order of magnitude higher and narrower than current state-of-the-art proposed GH sensing substrates with more complex nanostructures (e.g., quasi-bound states in the continuum and graphene, in which so far no experimental validation has been shown)^32,33^.

The relationship between phase singularity and reflectance is illustrated in Fig. 3c, d. It reveals two rules for engineering the sensing substrate. Firstly, the suppressed reflection will accelerate the phase change. An abrupt phase drift is expected at the minimal reflection point where the phase-changing rate is maximized. This phase drift will become a singular ${\boldsymbol{\pi }}$ jump when the reflectance is approaching zero as illustrated in Fig. 3c (silver-GST with 785 nm excitation). Secondly, narrowing the SPR curve also contributes to the enlarged phase changing rate by comparing two operating wavelengths (Also see Fig. S6). Such phase change enhancement is a result from the accelerated reflectance change near the minimal reflection point.

When the refractive index of the sensing medium is changed, the GH peaks will drift together with the resonance angle. Therefore, for a sensing substrate fixed at the initial resonance angle, the differential GH shift will vary according to the refractive index change of sensing medium. This response translates the sensitivity of the device. Both a higher peak value and a narrower peak width of differential GH shift led to a higher sensitivity. To quantify the device performance at different conditions, the figure of merit (FOM) was calculated for GH peaks as labeled in Fig. 3a, b. Herein, the FOM is determined by ($\Delta {GH}$/$\Delta n$)/${FWHM}$, in the unit of μm (RIU∙°)^−1^. $\Delta {GH}$ and $\Delta n$ denote the change in differential GH shift and the change in medium refractive index, respectively, while ${FWHM}$ is the full width half maximum of the peaks. The FOMs labeled in figure are calculated at $\Delta n=$10^−5^ ${RIU}$. The largest FOM was found on silver-GST substrate with 785 nm excitation as well, which is 4.54 × 10^11^ μm (RIU∙°)^−1^.

**Fig. 4:**
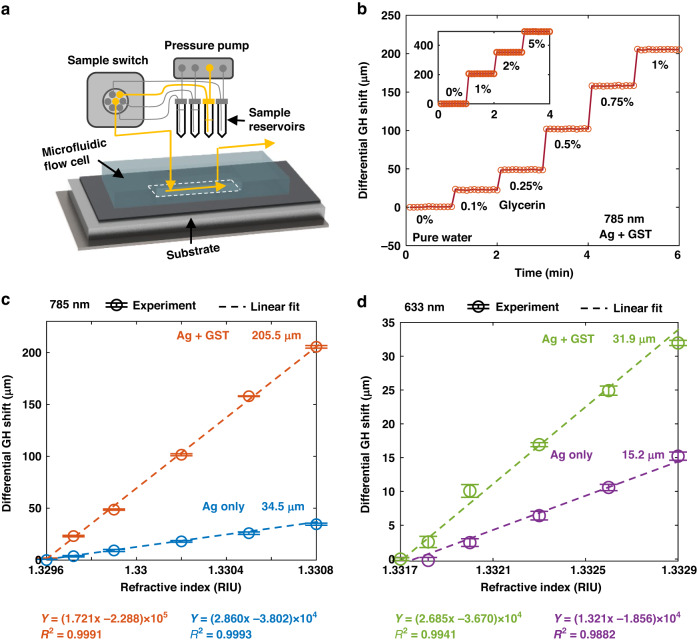
Sensitivity characterization of fabricated sensing substrates with glycerin solutions. **a** Schematic of the microfluidic system for loading the samples on sensing substrate. The active injection channel is marked in yellow. **b** The real-time measurements of GH shift induced by glycerin solutions on silver-GST substrate at 785 nm. **c**, **d** The sensor responses in GH shift to refractive index changes on both silver and silver-GST substrates at 785 nm (**c**) and 633 nm (**d**). The labeled value is the maximum shift measured with 1% glycerin solution. The sensor response was measured with the same batch of glycerin solutions. The error bar denotes the variation of GH shift in 3 repeated experiments

As illustrated in Fig. 4a, a microfluidic system was built to realize the batch measurement of samples for the sensitivity characterization and further biomarker targeting experiments. This system includes a microfluidic flow controller (OB1 MK4, Elveflow Inc., France), a switching valve (MUX DISTRIB, Elveflow Inc., France), a set of four sample reservoirs and a flow cell attached to the sensing substrate. The volume of microfluidic flow cell is 200 μL. The samples are selected by the switching valve installed between the sample reservoirs and the microfluidic flow cell. The injection pressure provided by the pump is 15 mbar.

Figure 4b gives an example of measuring a batch of glycerin solutions in water with gradient concentrations from 0.1% to 5% w/w. This measurement was conducted on the silver-GST substrate with 785 nm excitation. The differential GH shift was increased step by step during the measurement. The shift reading was linear with a glycerin concentration lower than 1% w/w (1.20 × 10^−3^), ranging up to 205.5 μm. The maximum GH shift over the linear range was 439.3 μm, measured by 5% of glycerin.

Figures 4c, d summarize the sensor response versus the refractive indices obtained by the measurement of glycerin solutions. The refractive index change induced by each 0.1% of glycerin is 1.20 × 10^−4^ RIU^34^. These calibration experiments with glycerin solutions are conducted on both silver and silver-GST substrate at 633 nm and 785 nm. Each measurement was repeated by 3 times. The sensitivity of each group can be obtained through the linear fitting of the calibration results, as plotted by dash lines in Fig. 4c, d. The group with higher peak of differential GH shift (Fig. 3a, b) was experimentally demonstrated to have a higher sensitivity. The sensitivity of silver-GST substrate operating at 785 nm was 1.72 × 10^8 ^nm RIU^−1^, is 10-times higher than the silver-GST substrate operating at 633 nm (1.32 × 10^7 ^nm RIU^−1^). This result, to the best of our knowledge^35,36^, is the first experimentally achieved sensitivity at level of 10^8 ^nm RIU^−1^.

### Detection of small biological molecules

**Fig. 5:**
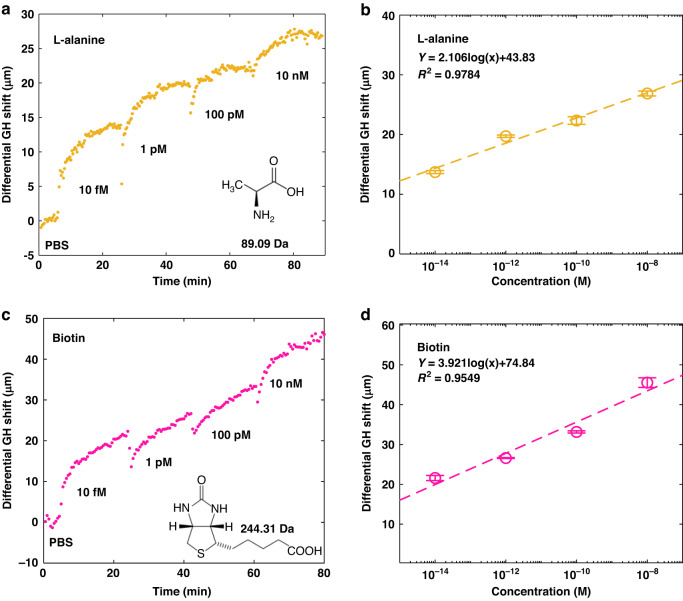
Detecting adsorbable small molecules. **a** Real-time measurement of l-alanine adsorption on silver-GST substrate. **b** The linear fitting of sensor response after 10 minutes of l-alanine adsorption. **c** The real-time adsorption measurement of biotin in PBS. **d** The linear fitting of sensor response after 20 min of biotin adsorption. These sensorgrams were acquired with 785 nm excitation. The error bar denotes the variation of GH shift measured at the last minute of adsorption measurement

It is challengeable for conventional angular-/intensity- interrogating SPR sensing schemes to detect small molecules (<1 kDa) with a concentration less than 1 pM^14–16^. In this work, we demonstrated the detection of L-alanine and biotin at a much lower concentration with silver-GST substrate operating at 785 nm. Since this demonstration was a directly adsorption of biomolecules, the new injection would flush away a few weakly bound molecules (especially for the low concentration ones). The sensor responses of serially diluted L-alanine in phosphate-buffered saline (PBS) from 10 fM to 10 nM are summarized in Fig. 5a, b. The differential GH shift after 20 min of 10 fM l-alanine adsorption was measured to be 13.7 μm. The differential GH shift induced by l-alanine increases by 2.106 μm for each order of magnitude increase in concentration.

The biotin test was also conducted with the same substrate and light source, as plotted in Fig. 5c, d. Different from the direct adsorption of L-alanine, the adsorption of biotin requires the functionalization of a positive linker, (3-Aminopropyl) trimethoxy silane (APTMS), on sensing substrate, because biotin is highly negative in neutral buffer due to the low isoelectric point of 3.5. The differential GH shift at 10 fM biotin was 21.6 μm, much higher than L-alanine due to the larger size. Furthermore, the linear fitting of biotin in Fig. 5d also showed a larger signal growth per concentration order of magnitude (3.921 μm) compared to l-alanine (2.106 μm), which is consistent with the heavier molar mass of biotin.

### Detection of cancer markers

**Fig. 6:**
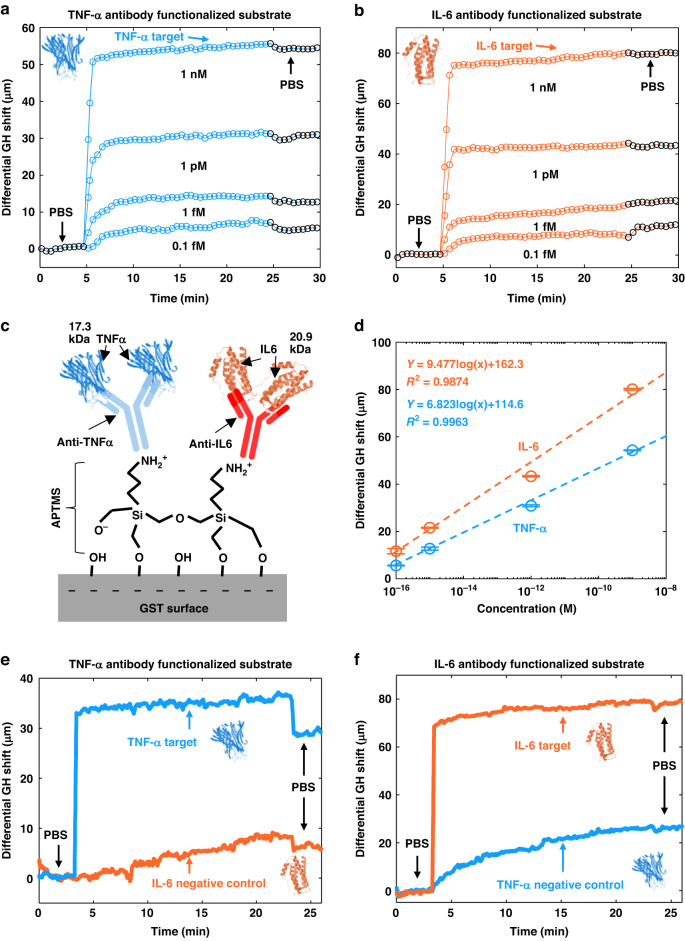
Specific cytokine detection with immobilized antibodies. **a**, **b** Sensorgrams showing the real-time detection of cancer markers TNF-alpha (**a**) and IL-6 (**b**) from 0.1 fM to 1 nM on silver-GST substrate at 785 nm. **c** The schematic of GST surface functionalization for cancer marker detection. **d** The comparison of sensor responses between TNF-alpha and IL-6 after 20 minutes of adsorption. The error bar denotes the variation of GH shift measured at the last minute of adsorption measurement. **e**, **f** Adsorption of non-specific and specific antigens on homologous antibodies**. e** The adsorption of TNF-α and IL-6 marker on TNF-α antibody functionalized sensor. **f** The adsorption of IL-6 and TNF-α marker on IL-6 antibody functionalized sensor

We have further demonstrated the detection of cancer markers to show the clinical potential of demonstrated biosensor. Two different cancer markers, including tumor necrosis factor alpha (TNF-α, 17.3 kDa) and interleukin-6 (IL-6, 20.9 kDa), were used in this targeting experiment. The measurement of cancer markers was based on the same silver-GST substrate for glycerin calibration and small molecule detection, operating at 785 nm. To obtain the stable binding of the cancer marker, a layer of the marker antibodies was immobilized on GST surface with APTMS (Fig. 6c). APTMS can anchor on the GST surface through the hydroxyl groups^37^, while the opposite amine end can promote the adsorption of antibodies^38^.

Figure 6a, b plot the sensorgrams of serially injected cancer markers from 0.1–1 fM. The cancer markers were incubated with the sensing substrate for 20 min and then flushed by PBS. At the lowest marker concentration of 0.1 fM, there were only 20 zeptomole of cancer markers injected inside considering the flow cell volume of 200 μL. The sensor response at this concentration after PBS flushing was 5.6 μm for TNF-α and 11.7 μm for IL-6. At a higher marker concentration, not only the signal shift increased after PBS flushing, but also the signal saturation time also became shorter. The signal rise continued over 5 min after the injection of 0.1 fM cancer markers. In contrast, the signal of 1 nM marker was saturated within 1 min after the injection.

The concentration of these two cancer markers in blood serum of a cancer patient is in the range of 20–50 pg mL^−1^ ^39–42^. Such concentration will be one order lower at early cancer stage II (in the order of 10^−13^ M)^40^. According to the linear fitting of IL-6 and TNF-α shown in Fig. 6d, the differential GH shift was measured of 39.1 μm for IL-6 and 25.9 μm for TNF-α at 10^−13^ M. Therefore, the demonstrated sensor satisfies the requirement for cancer diagnosis even at an early stage.

Additionally, we measured 1 nM non-specific and specific antigens on the substrate immobilized with homologous antibodies to validate the selectivity of analyte, as depicted in Fig. 6e, f. For IL-6 antibody, the sensor response from non-specific TNF-α adsorption (27.9 μm) was much lower than the specific IL-6 (77.6 μm). For TNF-α antibody, the sensor showed good contrast on these antigens as well. The non-specific antigen IL-6 led to a 11.6 μm shift while the specific TNF-α showed 28.8 μm larger shift. The sensor response shown in Fig. 6e is lower than 6f, which is due to the measurement starting at an incident angle with lower GH sensitivity. However, it is worth noting that in both tests, the signal induced by the non-specific binding has a consistent low ratio (~20%) to that of specific binding, indicating a significant selectivity of functionalized substrate.

### Monitoring the integrin affinity to RGD peptide

We also investigated the binding kinetics between integrin and RGD tripeptide to demonstrate the versatility of our silver-GST sensing substrate. The affinity of integrin is regulated by several factors, including but not limited to the concentration of divalent cations^43^, the mechanical tension of the cytoskeleton^44^, and the binding of regulatory proteins (cell contractility)^45^. In this demonstration, we focused on real-time monitoring of the affinity enhancement by increasing the Mn^2+^ concentration in buffer.

**Fig. 7:**
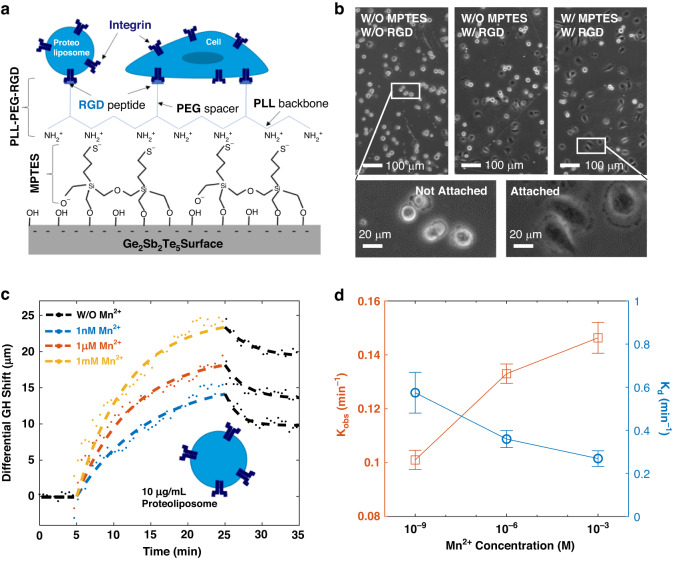
Monitoring the binding kinetics of integrin-embedded proteoliposomes to RGD peptide. **a** Schematic of GST surface functionalization for cancer marker detection. **b** Micrographs showing the attachment of fibroblasts on GST surface and RGD/MPTES functionalized GST surface. **c** The binding dynamic of integrin-embedded proteoliposomes on RGD functionalized silver-GST substrate, measured with different Mn^2+^ concentrations in buffer. The straight black dash line is the baseline of sensor response in Mn^2+^ free buffer. The colored dashed curves and the following black curves represent the exponential fits of the proteoliposome association and dissociation processes, respectively. **d** The observable binding constant (K_obs_, orange line) and the dissociation constant (K_d_, blue line) of integrin-embedded proteoliposomes to RGD peptide versus the concentration of Mn^2+^ ions. The error bar denotes the 95% confidence region of binding constant

Similar to the functionalization protocol used for immunoassays, the RGD grafted co-polymer PLL-PEG-RGD (PLL-g-PEG/PEG-RGD, SuSoS, Switzerland) was immobilized on GST top layer through a silane linker - (3-Mercaptopropyl) trimethoxy silane (MPTES; Figs. 7a and S4). Since the poly-L-lysine (PLL) backbone of PLL-PEG-RGD has a large number of positively charged amine groups, the negative thiol groups on immobilized MPTES will facilitate its adsorption on sensor surface. The adsorption of RGD grafted co-polymer on GST substrate, as well as the adsorption facilitation by MPTES, was confirmed by a cell spreading experiment. As depicted in Fig. 7b, three GST-coated substrates were incubated with fibroblasts (WPMY-1 cell) for half an hour. The top left one was untreated, the middle one was functionalized with PLL-PEG-RGD only, and top right one was processed with both MPTES and PLL-PEG-RGD. There were 82% of cells in average well spread on MPTES- and PLL-PEG-RGD-functionalized substrate, indicating a better functionalization quality than the substrate only treated with PLL-PEG-RGD (51%) and the untreated substrate (22%). Because the proportion of attached cells through integrin is positively correlated with the density of grafted RGD peptides.

The demonstration of Mn^2+^ induced affinity enhancement was based on the measurement of integrin-embedded proteoliposome instead of cells. By reconstituting purified α_IIb_β_3_ integrins into liposomes, we can isolate the integrins from the cellular environment and study the binding kinetics independently. Figure 7c gives the sensorgrams of the association and dissociation processes of 10 μg mL^−1^ proteoliposomes with different Mn^2+^ concentration from 1 nM to 1 mM in buffer. The increasing Mn^2+^ concentration facilitated the adsorption of proteoliposomes with a higher shifting curve. The association processes can be described by an exponential fit ${L}_{b}\bullet \left(1-{e}^{-{k}_{{obs}}t}\right)$, while the dissociation process is$\,{L}_{b}\bullet {e}^{-{k}_{d}t}+{L}_{d}$. Here ${L}_{b}$ and ${L}_{d}$ denote the GH shifts after proteoliposome binding and dissociation. ${k}_{{obs}}$ and ${k}_{d}$ are observable association constant and the dissociation constant, respectively. Figure 7d illustrates the ${k}_{{obs}}$ and ${k}_{d}$ of proteoliposomes versus the Mn^2+^ concentration. With increasing ion concentration, the ${k}_{{obs}}$ was increased from 0.101 min^−1^ to 0.146 min^−1^ and ${k}_{d}$ was decreased from 0.574 min^−1^ to 0.269 min^−1^. It means the proteoliposomes adsorption became faster while the desorption was slowed, indicating a higher affinity of embedded integrins.

## Discussion

This work demonstrated a SPR biosensing platform based on the interrogation of the giant GH shift. Such shift is generated from the singular phase retardation at the darkness point of reflection on resonating plasmonic substrate. A hybrid sensing substrate of atomically thin GST on silver nano layer was designed and fabricated to obtain the quasi-zero (1.03 × 10^−6^) reflectance at resonance. The advanced performance in reflectance suppression is resulted from the extra absorption of the designed 1-nm thin GST layer. As given in Fig. 2, a larger absorption both in measurement and calculation was demonstrated on GST deposited substrates. Besides, as illustrated in Fig. S5, such absorption is not available by varying the thickness of bare silver substrates. Consequently, due to this improved darkness in reflectance, the phase response on silver-GST substrate was topologically singularized (Fig. 3a, c).

To quantitively compare the GH sensing response among different substrates and sensing conditions, the FOM defined by ${senstivity}/{FWHM}$ was introduced with the unit of μm (RIU∙°)^−1^. As abovementioned, lowering the minimum reflectance is the main factor leading to higher FOM as it brings the phase singularity. But in addition, narrowing the reflectance curve has also contributed to the FOM enhancement. This effect is observable by comparing the reflectance curve between 633 and 785 nm, especially for that with similar minimum reflectance at same order of magnitude. (Figs. 3 and S6, and Table S1). It is due to the accelerated reflectance change near the darkness point, that enlarges the phase changing rate as well. Through the enhancement of these two factors, the FOM of silver-GST substrate operating at 785 nm was significantly magnified to 4.54 × 10^11^ μm (RIU∙°)^−1^.

The experimentally measured sensitivity on optimized substrate was 1.72 × 10^8 ^nm RIU^−1^, which is the highest one obtained on phase-singularity based plasmonic sensors^35,36,46^. Considering the 0.12 μm noise level, the detection limit in refractive index is determined to be 6.97 × 10^−7^ RIU. This result is one order of magnitude better than recently developed interferometric setups based on microarrays and grating nanogrooves for practical diagnostics^47–49^. The position detector used in this work is cost effective with moderate performance. It is also worth noting that our detection limit can be further improved to the order of 10^−9^ and even 10^−10^ RIU by using a high-resolution beam position detector^50^ (the position resolution is <1 nm) and combined with optical magnification.

The biosensing performance of our platform was first evaluated with small molecules that have molecule mass <1 kDa. Our sensor is able to detect the free absorption of l-Alanine (89.09 Da) with a minimal concentration of 10 fM and achieved a GH shift of 13.7 μm. As for the adsorption of negatively charged biotin molecules, the sensing substrate was functionalized with positively charged APTMS for better adsorption. The demonstrated minimal detectable concentration is also 10 fM, but that led to a higher GH shift of 21.6 μm.

To further evaluate the potential of trace molecule detection, we functionalized the sensing substrate with antibodies that enabled the specific binding of targeting molecules at lower concentration. The demonstration used TNF-α (17.3 kDa) and IL-6 (20.9 kDa), both were detectable at a concentration of 0.1fM or equivalent to 20 zeptomoles molecules in flow chamber, resulting in 5.6 μm and 10.7 μm of GH shift, respectively. Due to the 0.12 μm resolution, the developed sensing platform has potential to detect sample with sub-zeptomole molecules inside. To the best of our knowledge, it is the first label-free demonstration of detecting such low concentration of analytes on plasmonic sensors (Tables S3 and S4).

In addition to the detection of molecules, we also assessed the binding kinetics between integrins and RGD peptides based on integrin-embedded proteoliposomes. The versatility of our sensing substrate has been validated with another saline linker MPTES. Opposite to APTMS, MPTES is negatively charged from the thiol group. The incubation of MPTES allows the better immobilization of positively charged molecules such as PLL-PEG-RGD, that has been demonstrated with a cell attachment experiment.

Overall, our plasmonic sensor based on singular phase interrogation offers an ultrasensitive biosensing at zeptomole level in real-time. It has great potential in both early disease detection and basic biological research that particularity requires an ultra-high sensitivity. In future work, we will further engineer the effective refractive index of sensing substrate to fine tune its optical response, which could lead to a better match of phase singularity. For instance, on amorphous GST layer, the partial crystallization with laser can produce specific patterns that modulate the propagation of surface plasmon wave. It will not only bring a higher sensitivity but also a more flexible sensing substrate design.

## Materials and methods

### Chemicals

Glycerin, Absolute Ethanol, Sodium Chloride, Bovine Serum Albumin (BSA), L-Alanine, D-Biotin, (3-Aminopropyl) trimethoxy silane (APTMS), (3-Mercaptopropyl) trimethoxy silane (MPTES), Interleukin-6 (IL-6), IL-6 antibody, tumor necrosis factor alpha (TNF-α), and TNF-α antibody were purchased from Sigma-Aldrich, France. Phosphate-buffered saline (PBS), Dulbecco’s Modified Eagle Medium (DMEM), N-2-hydroxyethylpiperazine-*N*-2-ethane sulfonic acid (HEPES), Fetal bovine serum (FBS), Glutamine, and Trypsin/EDTA solutions were purchased from Thermo-Fisher, France. Ultrapure water was purified by a PURELAB Flex system purchased from ELGA, UK. Arginylglycylaspartic acid grafted poly-l-lysine-graft-poly (ethylene glycol) (PLL-g-PEG/PEG-RGD) was purchased from SuSoS, Switzerland.

### Bio-sample preparation

The glycerin solutions were prepared in ultrapure water with concentrations of 0.1%, 0.25%, 0.5%, 0.75%, and 1% w/w. The l-Alanine and d-Biotin powder were prepared as 0.1 mM stock solutions and filter sterilized by a 0.2-μm syringe filter. They were further made into serial dilutions from 10 fM to 10 nM with a dilution ratio of 1:100 for measurement. APTMS with a concentration of 1 mM was prepared in PBS. MPTES was dissolved in absolute ethanol to 1 M and sequentially diluted in PBS to 1 mM. BSA powder was dissolved in PBS to a concentration of 1 μM and filter sterilized by a 0.2-μm syringe filter. IL-6 and TNF-α powder were reconstituted in ultrapure water to a stock concentration of 0.5 mg mL^−1^. The stock solutions of these two markers were further diluted with PBS to 0.1 fM, 1 fM, 1 pM, and 1 nM for measurement. The antibodies of IL-6 and TNF-α were purchased as reconstituted stock solutions with a concentration of 0.5 mg mL^−1^. The stock solutions were further diluted to 10 pM. The fibroblast culture medium was DMEM with 0.01% FBS (v/v), 20 μM Glutamine and 400 μM HEPES. The PLL-PEG-RGD was diluted in ultrapure water to 0.15 mg mL^−1^ with 10 mM HEPES and 150 mM NaCl. The running buffer for measuring the proteoliposome adsorption was the 1:10 dilution of PBS.

### Surface functionalization

In this work, the top GST layer on silver-GST substrate was functionalized with saline linkers, such as APTMS and MPTES. The substrate was rinsed with absolute ethanol and ultrapure water first. Rinsed substrate was dried in nitrogen flow. The substrate was mounted with a flow cell after cleaning. For D-biotin detection, 1 mM of APTMS was injected inside and incubated with the substrate for at least one hour. For the specific binding of cancer marker, 10 pM of antibodies were further incubated for 30 min to obtain the immunoactivity. For RGD functionalization, 1 mM of MPTES was first incubated with the substrate for 1 h. After that, 0.15 mg RIU^−1^ PLL-PEG-RGD was incubated for one hour. The RGD peptide grafted on PLL backbone can induce the cell attachment with integrins on cell membrane.

### Supplementary information

Supplementary Material
