# Supplementary material for: Label-free biosensing with singular-phase-enhanced lateral position shift based on atomically thin plasmonic nanomaterials
Source: Light Sci Appl. 2024 Jan 1;13:2. doi: 10.1038/s41377-023-01345-6 (PMC10757996; doi:10.1038/s41377-023-01345-6)
Supplement: Supplementary file 1 — Supplementary Material [file 41377_2023_1345_MOESM1_ESM.pdf]

Supplementary information for "Label-free biosensing with singular-phase-enhanced lateral position shift based on atomically thin plasmonic nanomaterials"

Shaodi Zhu[1,2], Rodolphe Jaffiol[1], Aurelian Crunteanu[3], Cyrille Vézy[1], Sik-To Chan[1,2], Wu Yuan[2], Ho-Pui Ho[2,*], Shuwen Zeng[1,*].

[1] Light, Nanomaterials & Nanotechnologies (L2n), CNRS-EMR 7004, University of Technology of Troyes, 10000 Troyes, France.

[2] Department of Biomedical Engineering, The Chinese University of Hong Kong, Shatin, New Territories, Hong Kong, China.

[3] XLIM Research Institute, UMR 7252 CNRS/University of Limoges, 123, Avenue Albert Thomas, Limoges, France.

*E-mail: aaron.ho@cuhk.edu.hk; shuwen.zeng@cnrs.fr.

Contents

Supplementary note 1: Characterization of the GST coating 2

Supplementary note 2: Substrate stability in solution 3

Supplementary note 3: Characterization of the system-noise 5

Supplementary note 4: Sensorgram during the functionalization 6

Supplementary note 5: Mechanism of the sensitivity enhancement by GST deposition 7

Supplementary note 6: Sensing performance comparison between gold and silver substrates . 9

Supplementary note 7: Theoretical model 11

Supplementary note 8: Refractive indices for transfer matrix calculation 12

Supplementary note 9: Performance analysis across different devices 12

Supplementary note 1: Characterization of the GST coating

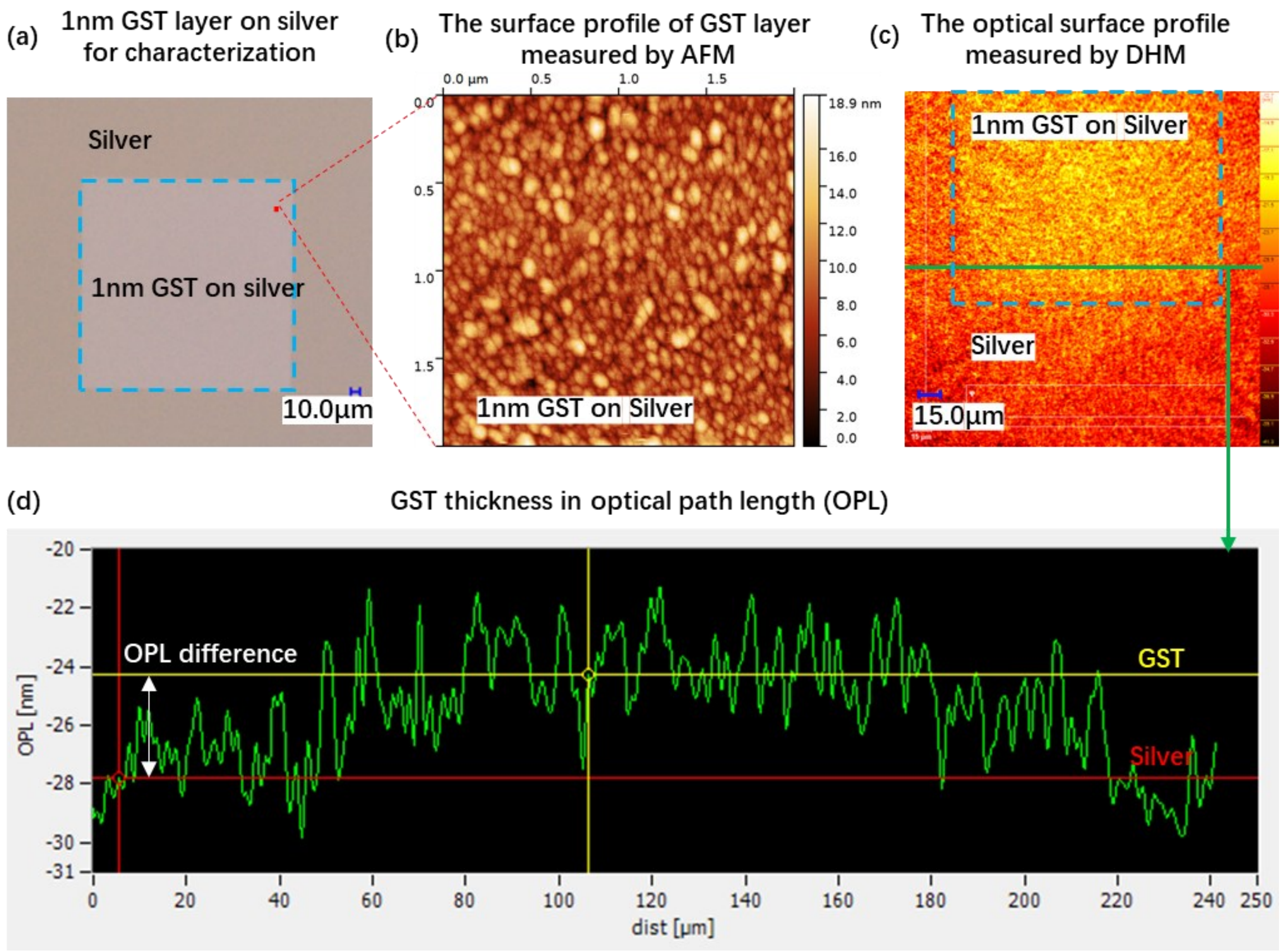

Supplementary Fig. S1 Characterization of the GST coating. a Micrograph of the silver-GST substrate for characterization. The GST covered region is marked by blue box. **b** The surface profile of GST covered region on substrate, measured by Atomic Force Microscope (AFM) in tapping mode. **c** The optical surface profile of fabricated silver-GST substrate, measured by Digital Holography Microscope (DHM). Blue box marks the GST covered region. **d** A cross-section view of the optical surface profile at the green line in figure **c**. X-axis is the distance to left starting point. Y-axis is the optical path length (OPL) determined by the real surface profile and material refractive index.

Fig. S1a shows the silver-GST substrate for characterization, which has the same thickness configuration as fabricated for sensing. The $Ge_2Sb_2Te_5$ deposition was performed using DC magnetron sputtering from a stoichiometric target, in Ar atmosphere. The Ar flow rate was set to 60 sccm (standard cubic centimeter per minute) for a total deposition pressure of 5×10^{-3} mbar. The calibration of layers thicknesses under a constant sputtering power of 20 W was performed for different sputtering times, resulting in a mean GST deposition rate of 11 nm/ min. The GST layer out of the blue box was removed by lift-off process. Fig S1b depicted the surface profile of a 2 µm × 2 µm area in GST covered region,

measured by the tapping mode of Atomic Force Microscope (Nanoscope, Bruker Inc., France). The mean surface roughness (Ra) in this region is 0.70 nm. Fig. S1c provides a wider optical surface profile for characterizing the thickness of GST capping layer, measured by Digital Holography Microscope (R2100, Lyncée Tec Inc., France). The optical profile is the optical path length distribution (OPL) on surface, which is determined by $T \times n_{material}$. T represent the real surface profile while $n_{material}$ denotes the material refractive index at the microscope working wavelength (675nm). Fig. S1d is a cross-section view of the optical profile illustrating the GST layer thickness measurement. The mean OPL difference between the top surface of GST capping and surround silver ranges from 3.53 to 4.10 nm over 10 profiles. The GST refractive index at 675nm is 3.93 [S1]. Therefore, the thickness of GST layer should be 0.90 to 1.04 nm.

Supplementary note 2: Substrate stability in solution

An immersion test in PBS was applied to validate the stability of bare silver substrate and the silver substrate with 1nm GST capping layer. In Figs. S2a and S2b, the GST capped region did not show observable degeneration after 1 hour and 24 hours of immersion, while the bare silver region was visibly prone to oxidation darkening with dense black spots (Fig. S2b). Moreover, Figs. S2c and S2d provide the reflectance decay after the immersion test to quantitively evaluate the degeneration on substrates. The decay curves were calculated by subtracting the after-immersion spectrum to raw spectrum. After 24 hours of immersion, the reflectance on silver substrate has reduced by 60%. In contrast, there was no observable decay on silver-GST substrate. Such enhanced stability of sliver-GST substrate can ensure the sensor performance during the measurement as the time for a single test (Fig. 6, 7) is around half an hour.

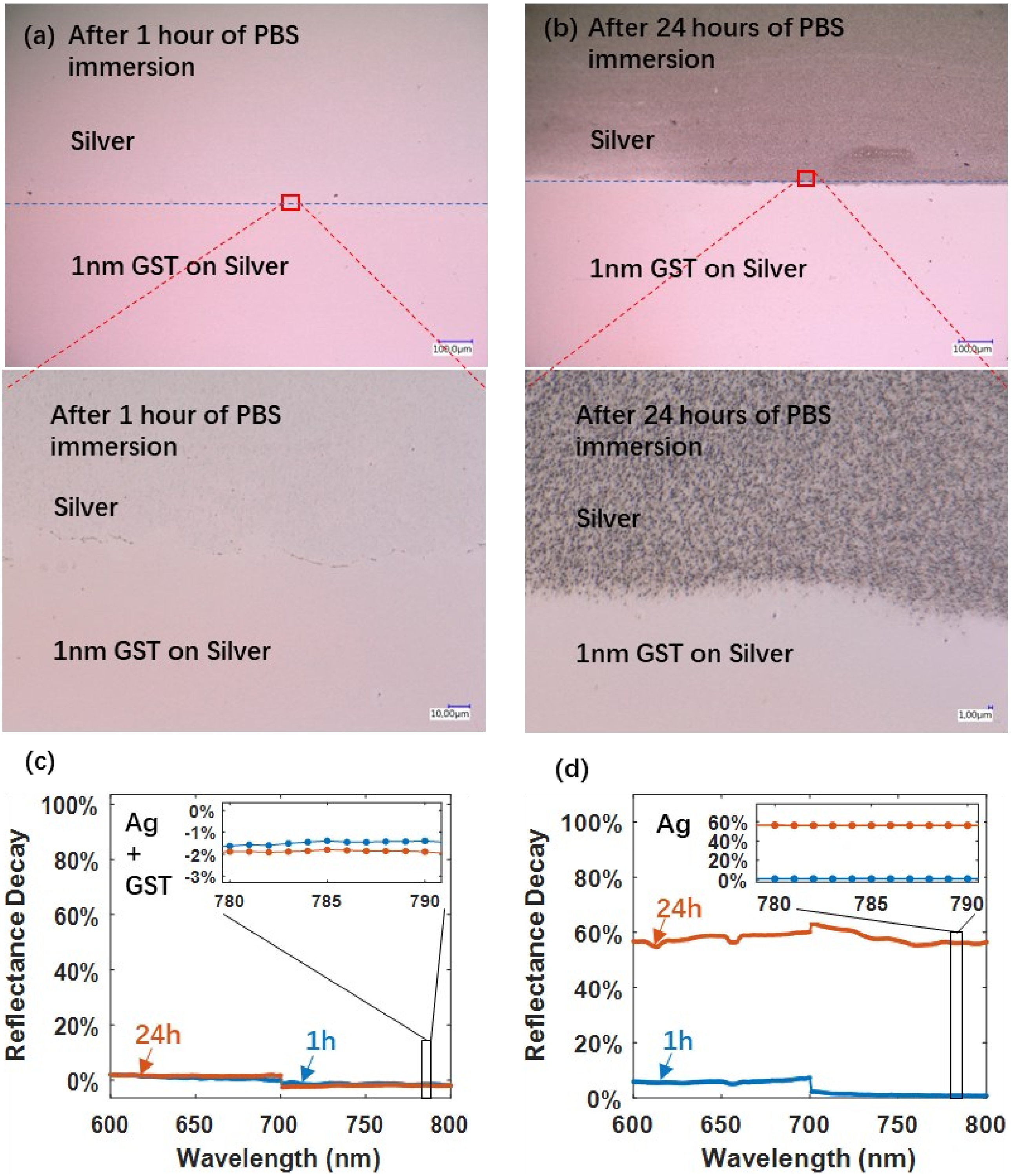

Supplementary Fig. S2 a, b Bright field micrographs of bare silver and 1nm GST coated silver substrate after 1- (**a**) and 24-hours (**b**) immersion in PBS buffer. The pictures are taken by digital microscope. **c, d** Reflectance decay measured on (**c**) GST coated silver (**d**) bare silver substrate after immersion test. The decay is measured by spectrometer in comparing with the spectrum acquired before immersion. Zoom in subplots inside illustrate the reflectance decay around the operating wavelength of 785nm. The negative value shown in (**c**) is due to the enlarged reflectance after buffer immersion and rinse. The spectrum shown in Figs S2c and S2d have a sudden drift at 700nm. It results from the misalignment of the dual sensors in spectrometer and independent to reflectance measurement.

Supplementary note 3: Characterization of the system-noise

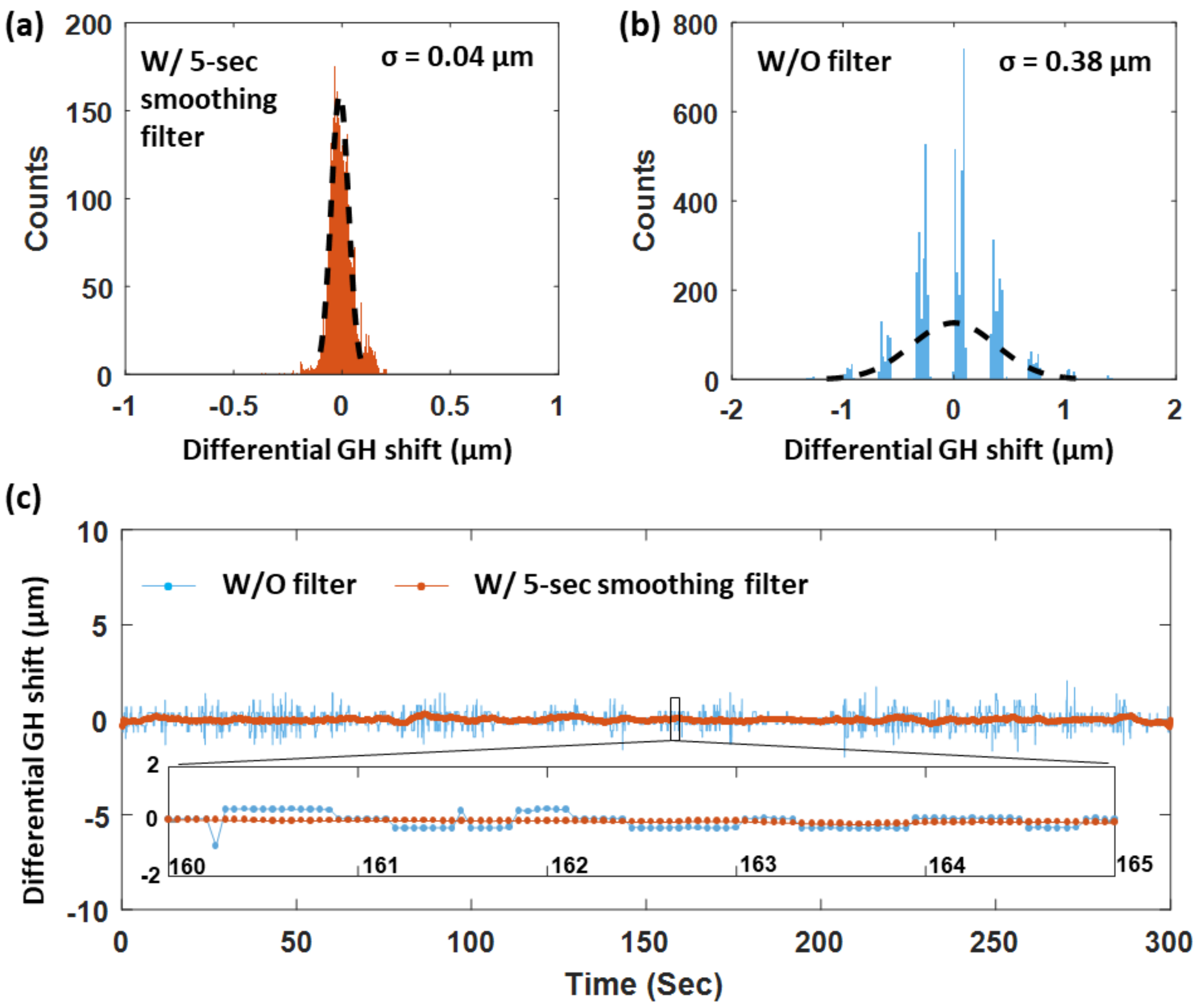

Supplementary Fig. S3 **a** The histogram of dataset acquired with deionized water. The dataset has 6000 data points recorded within 300 seconds (20 Hz). The black dash line denotes the fit of normal distribution. A 5-second moving average filter was applied to remove high frequency noise. **b** The histogram of raw dataset. The discrete peaks are due to the analog to digital conversion. **c** Raw sensorgram (blue) and smoothed (orange) sensorgram plotted from the dataset. The zoom-in figure inside provides the sensorgram detail from 160 s to 165 s.

Fig. S3 provides the characterization of system noise using deionized water. The data set has 6000 data points within 300 seconds (20 Hz). As depicted in Figs. S3a and S3b, the normal fit of raw data distribution has a standard deviation of 0.38 μm. Due to the analog-to-digital converter, the data distribution has multiple discrete clusters inside. After applying a 5-second smoothing filter, the standard deviation will be significantly reduced to 0.04 μm. The sensorgrams are plotted in Fig S3c as the reference. According to Fig S3b, the experimental noise level (3σ) should be 0.12 μm.

Supplementary note 4: Sensorgram during the functionalization

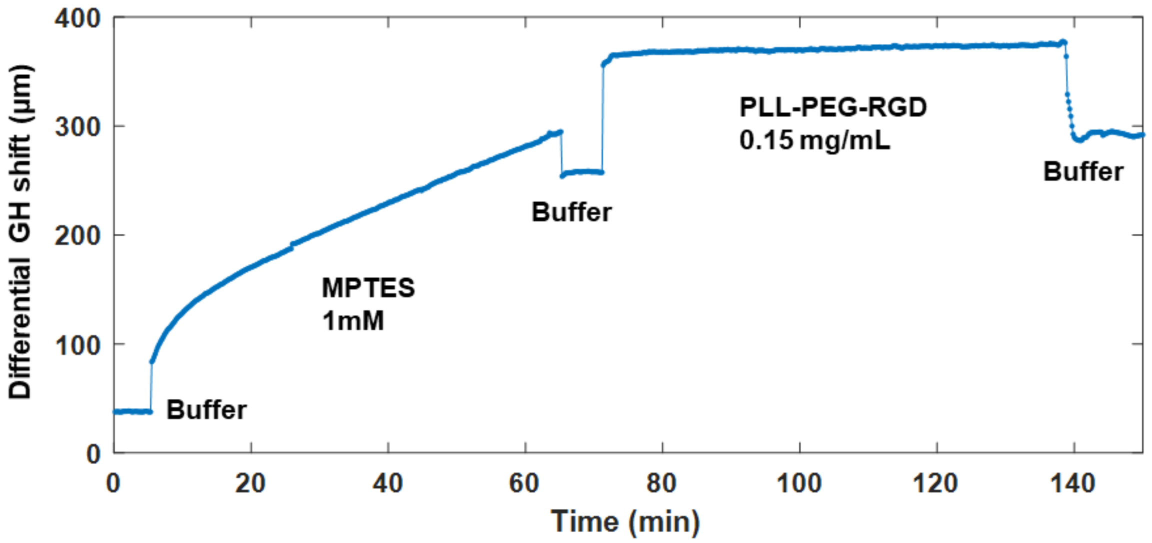

Supplementary Fig. S4 The sensor response during the functionalization for proteoliposomes adsorption as illustrated in Fig. 7a.

Fig. S4 shows the raw sensorgrams plotted in 10 second per point during the functionalization procedure for measuring the integrin-embedded proteoliposome. The incubation of MPTES and subsequent buffer flushing resulted a GH shift of 267.7 μm. The further adsorption of PLL-PEG-RGD showed a 294.3 μm shift after buffer flushing. Because the total GH shift has suppressed the linear region of sensor after the adsorption of MPTES, the adsorption curve of PLL-PEG-RGD adsorption was saturated and not as steep as the MPTES curve.

Supplementary note 5: Mechanism of the sensitivity enhancement by GST deposition

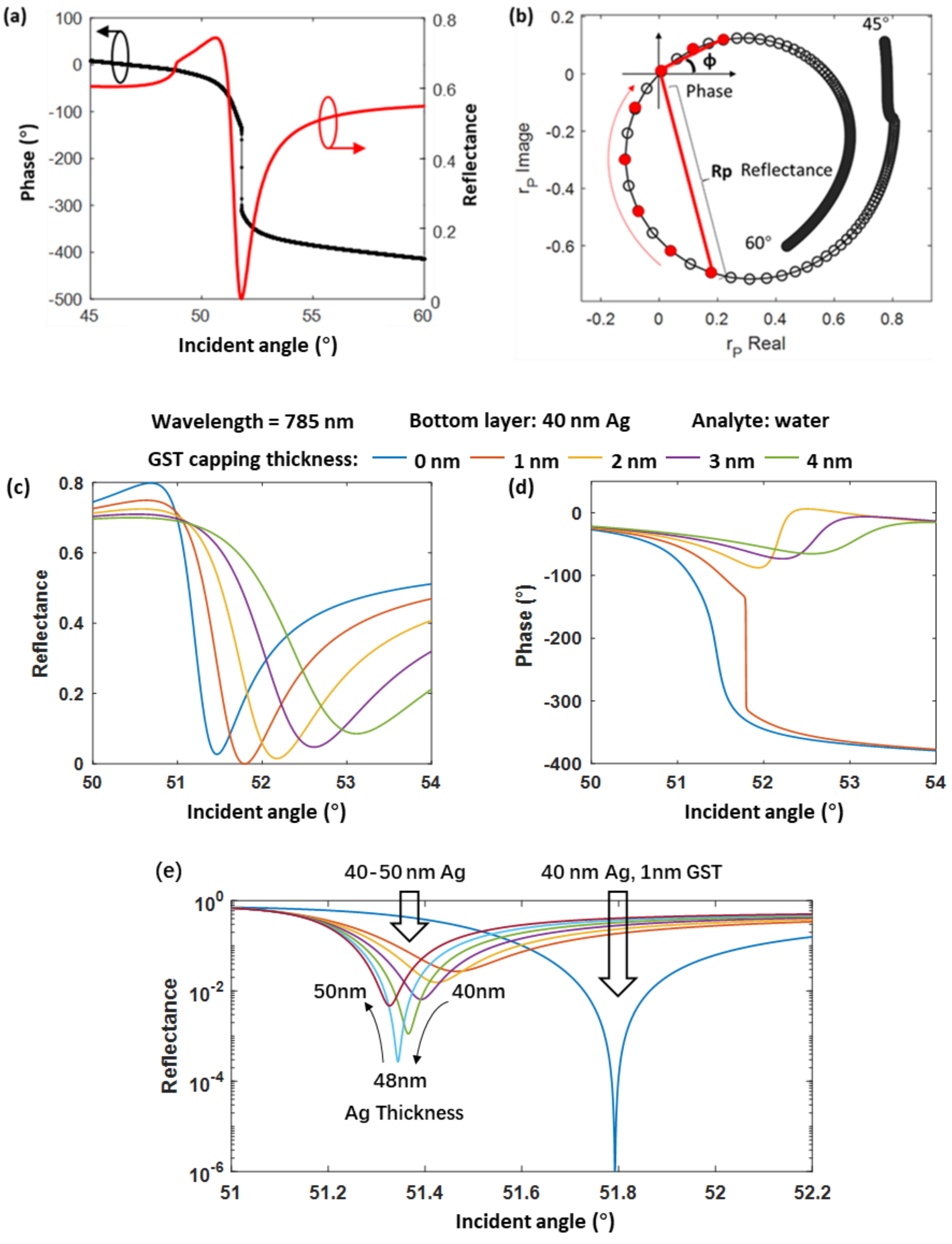

Supplementary Fig. S5. a The calculated reflectance and phase curve of p-polarized incident light on silver-GST sensing substrate. The incident angle was scanned from 45° to 60° with a step size of 0.01°. **b** The calculated complex reflection coefficient r_p mapped on complex plane as the function of incident angles. The X and Y axes represent the real and image parts of r_p, respectively. R_p as the norm of r_p denotes the reflectance. Φ as the phase angle of r_p denotes the phase of incident light. The angle scanning range and step are same as Fig. **a**. **c, d** Reflectance comparison (**c**) and phase comparison (**d**) for silver substrates with varying GST coating. **e** Reflectance comparison between silver substrates and optimized silver-GST substrate.

The relationship between reflectance and phase is illustrated in Fig. S5a. The phase changing rate will be accelerated if the reflection is suppressed. At the minimal reflection point, the phase changing rate is maximized, resulting in an abrupt phase jump. Such abrupt phase change can be topologically explained on a complex plane as depicted in Fig. S5b. The X and Y axes of this plane respectively represent the real and image parts of complex reflection coefficient r_p. R_p as the norm of r_p denotes the reflectance. Φ as the phase angle of r_p denotes the phase of incident light. If r_p vector is approaching the origin on complex plane, i.e., $R_p \to 0$, the change rate of phase angle will be accelerated that eventually results in an abrupt jump. Furthermore, according to equation S1: $\Delta GH = -\frac{\lambda_1}{2\pi n_1}\left(\frac{\partial\phi_p - \partial\phi_s}{\partial\theta}\right)$, the sharpened phase jump brings a larger GH shift and higher sensitivity. Therefore, the suppressed reflection is the direct factor leading to better GH sensing performance.

The 1nm GST layer was employed to further minimize the reflectance at the resonance angle of the sensing device. Since GST alloy is an absorptive material in visible to NIR range [S2,S3]. As given in Figs. S5c and S5d, such GST layer thickness is designed to avoid the over flatten of reflectance curve and subsequent mismatching of phase singularity condition. Also, it is not possible to use metallic layer only to achieve this maximized light absorption. We plotted the silver substrate response by tuning the thickness as shown in Fig. S5c. The minimum reflectance is 2.69×10^{-4}, not as low as the result achieved by silver-GST substrate (1.03×10^{-6})

Supplementary note 6: Sensing performance comparison between gold and silver substrates

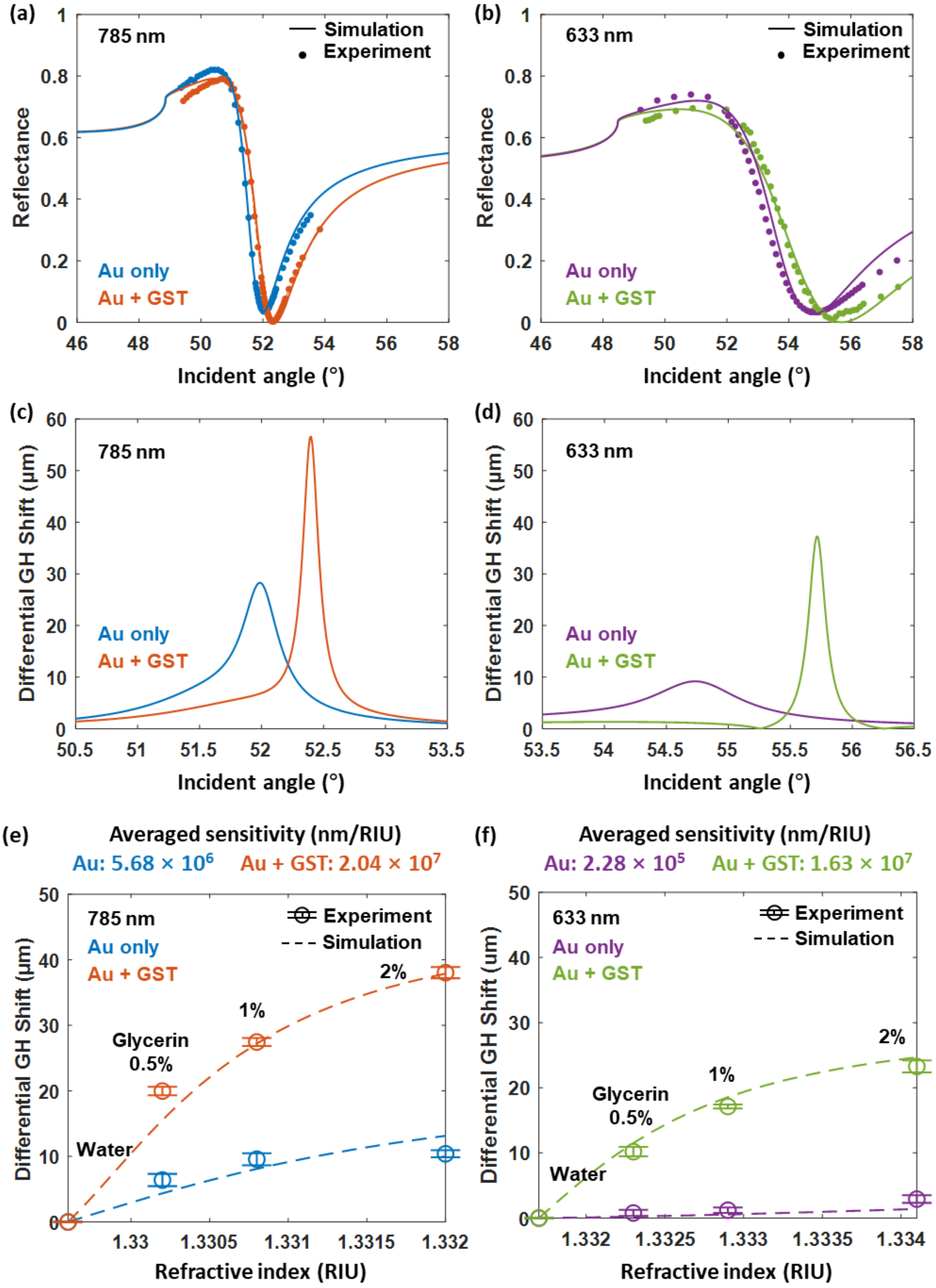

Supplementary Fig. S6 Characterization of gold and gold-GST sensing substrate. a, b The experimental and calculated reflectance curves of gold and gold-GST substrates at 785 nm (**a**) and 633 nm (**b**). **c, d** The calculated differential GH shifts versus incident angle at 785 nm (**c**) and 633 nm (**d**). **e, f** The sensor responses in differential GH shift to refractive index changes at 785 nm (**e**) and 633 nm (**f**). Sensing substrates are tested in deionized water and glycerin solutions with different concentrations (0.5%, 1% and 2% w/w). The simulated sensor response is marked by dash line as reference.

Table S1: Summary table of sensing performance related parameters between different substrates and operating wavelengths

Substrate	λ	Minimal reflectance (a.u.)	FWHM of SPR dip (°)	maximum GH Shift (μm)	FWHM of GH peak (°)	GH sensing FOM (μm (RIU·°)$^{-1}$)
**Silver-GST**	**785nm**	**1.03×10^{-6}**	**1.58**	**7930.4**	**0.001**	**4.54×10^{11}**
(Fig 2,3)	633nm	2.32×10^{-3}	3.09	42.0	0.135	4.01×10^{4}
Silver	785nm	2.75×10^{-2}	1.31	57.7	0.203	4.30×10^{4}
(Fig 2,3)	633nm	7.61×10^{-2}	2.02	17.5	0.721	9.58×10^{2}
Gold-GST	785nm	4.16×10^{-3}	5.99	56.6	0.185	1.24×10^{5}
(Fig S6)	633nm	4.96×10^{-4}	8.27	37.3	0.170	9.98×10^{4}
Gold	785nm	3.45×10^{-2}	2.37	27.9	0.433	1.30×10^{4}
(Fig S6)	633nm	2.59×10^{-2}	2.96	9.1	1.044	2.16×10^{3}

As shown in Fig. S6, in order to do a better comparison between silver substrates and conventional gold substrates. we have fabricated the gold sensing substrates with similar thickness parameters. In Figs S6a and S6b, the reflectance matching results indicate that the fabricated substrates have 38nm Au and 1nm GST. As we can see for both 785 nm and 633 nm, the GH width curve and the experimentally measured sensitivity all have a similar trend as for the silver substrates. For example, in Figs. S6c and S6d, the maximum GH shift of 56.6 μm were found on GST coated substrate with 785 nm excitation as well. However, compared to the silver-GST substrate operated at same wavelength, the decreased peak value of GH shift on gold-GST substrate resulted in nearly 10 times lower experimental sensitivity as shown in Figs. S6e, S6f and 4c, 4d.

The sensing performance related parameters are listed in Tabel S1, for the comparison between different sensing substrates and sensing conditions. To quantitively compare the GH sensing response, the figure of merit (FOM) defined by sensitivity/FWHM was introduced in this table with the unit of μm (RIU·°)$^{-1}$. The gold-GST sensing substrate operated at 785 nm has a FOM value of 1.24×10^{5}, which was much lower than that of silver-GST substrate (4.54×10^{11}).

Supplementary note 7: Theoretical model

The length of differential Goos-Hänchen shift ΔGH can be calculated by the stationary phase theory:

$$\Delta GH = -\frac{\lambda_1}{2\pi n_1}\left(\frac{\partial \phi_p - \partial \phi_s}{\partial \theta}\right) \tag{1}$$

Where n_1 is the refractive index of prism. λ_1 denotes the wavelength of incident light. θ and ϕ are the incident angle and the phase shift of reflection, respectively. The subscripts p and s denote the polarization of incident light.

The reflectance R and the phase shift of reflection ϕ are functions of incident angle θ, calculated from the complex reflection coefficient r by Fresnel equations:

$$\phi = \arctan\frac{Im\left(r_{p/s}(\theta)\right)}{Re\left(r_{p/s}(\theta)\right)} \tag{2}$$

$$\mathrm{R} = \left|r_{p/s}(\theta)\right| \tag{3}$$

For a multi-layered structure, the complex reflection coefficient $r_{p/s}(\theta)$ can be derived from transfer matrix method. The characteristic transfer matrix $M$of order 2 with N total layers is:

$$M = \prod_{i=2}^{N-1} M_i \tag{4}$$

$$r_{p/s}(\theta) = \frac{(M_{11} + M_{12}q_{Np/Ns})q_{1p/1s} - (M_{21} + M_{22}q_{Np/Ns})}{(M_{11} + M_{12}q_{Np/Ns})q_{1p/1s} + (M_{21} + M_{22}q_{Np/Ns})} \tag{5}$$

Where for non-magnetic substance:

$$M_{ip/is} = \begin{bmatrix} \cos(\beta_{ip/is}) & -i\sin(\beta_{ip/is})/q_{ip/is} \\ -i\sin(\beta_{ip/is})q_{ip/is} & \cos(\beta_{ip/is}) \end{bmatrix} \tag{6}$$

$$\beta_{ip/is} = 2\pi t_i/\lambda_1 \tag{7}$$

$$q_{ip} = \sqrt{n_i^2 - n_1^2\sin^2\theta}\,/(n_i^2) \tag{8}$$

$$q_{is} = \sqrt{n_i^2 - n_1^2\sin^2\theta} \tag{9}$$

Supplementary note 8: Refractive indices for transfer matrix calculation

Table S2. List of Refractive indices for transfer matrix calculation

Material	633nm	785nm
**SF11**	1.7786	1.7655
**Sapphire**	1.7659	1.7605
**Titanium** [S4]	2.1526 + 2.9241i	2.7920 + 3.3120i
**Silver** [S5]	0.0590 + 3.9402i	0.0850 + 5.1360i
**GST** [S1]	3.8732 + 1.4408i	4.0481 + 1.0014i

Supplementary note 9: Performance analysis across different devices

Table S3. Comparison of biosensing performance with commercial products

Ref	Instrument	Analyte (with molar mass)	Contrast agent	Lowest analyte concentration demonstrated
This work	self-build	TNF-α (17.3 kDa)<br>IL-6 (20.9 kDa)<br>L-alanine (0.089 kDa)<br>Biotin (0.244 kDa)	No need for signal amplification with contrast agents **(label-free)**	1×10^{-4} pM<br>1×10^{-4} pM<br>1×10^{-2} pM<br>1×10^{-2} pM
S6	Biacore 2000	Testosterone (0.288 kDa)	gold nanoparticles	3.7 pg mL^{-1} (12.8 pM)
S7	Biacore 2000	Progesterone (0.315 kDa)<br>Progesterone (0.315 kDa)	gold nanoparticles<br>No contrast agent	8.6 pg mL^{-1} (28.3 pM)<br>35-60 pg mL^{-1} (115-200 pM)
S8	Biacore Q	Progesterone (0.315 kDa)	gold nanoparticles	4.9 pg mL^{-1} (15.6 pM)
S9	NanoSPR-321	Nitroglycerin (0.227 kDa)	gold nanoparticles	20 pM
S10	NanoSPR-321	Neomycin (0.615 kDa)<br>Streptomycin (0.582 kDa)	gold nanoparticles<br>gold nanoparticles	2.0 pM<br>0.2 pM
S11	Biacore 3000	Cardiac myoglobin (17 kDa)	gold nanoparticles	10 pM
S12	Biacore x	Thrombin (37 kDa)	gold nanoparticles	100 pM
S13	Autolab SPR system	a-Fetoprotein (70 kDa)	Fe_3O_4–gold nanoparticles	650 pg mL^{-1} (9.3 pM)

Table S4. Comparison of biosensing performance with previous works

Ref	Sensing technique	Measured quantity	Sensing substrate	Analyte (With molar mass)	Lowest analyte concentration demonstrated
This work	plasmon-enhanced Goos-Hänchen effect	position shift	silver layer + ultrathin GST	TNF-α (17.3 kDa)<br>IL-6 (20.9 kDa)<br>L-alanine (0.089 kDa)<br>Biotin (0.244 kDa)	1×10^{-4} pM<br>1×10^{-4} pM<br>1×10^{-2} pM<br>1×10^{-2} pM
27	ellipsometry	phase shift	dual gold nanodots array + graphene	streptavidin (60 kDa)	10 pM
25	ellipsometry	phase shift	asymmetric metal-dielectric cavity	streptavidin (60 kDa)	1 pM
S14	surface plasmon resonance (SPR)	wavelength spectrum of absorption	gold/Al_2O_3 multilayers	BSA (66.5 kDa)<br>Biotin (0.244 kDa)	1×10^{-2} pM<br>10 pM
S15	plasmon-enhanced fluorescence	wavelength spectrum of emission	gold nanoparticle array	PfLDH malaria biomarker (34.9 kDa)	1×10^{-2} pM
S16	Localized SPR imaging	transmission Intensity	gold nanohole array	IgG (150 kDa)	3.9μg mL^{-1} (26×10^{3} pM)
S17	interferometric microarray imaging	optical path difference	gold nanohole array	secondary IgG (150 kDa)	0.5μg mL^{-1} (9×10^{3} pM)
S18	tilted fiber Bragg grating (TFBG) based SPR	wavelength spectrum of absorption	gold coated TFBG	endocrine disruptor (36.8 kDa)	0.01 ng mL^{-1} (0.27 pM)
S19	surface plasmon resonance (SPR)	wavelength spectrum of absorption	gold nanorod array	Biotin (0.244 kDa)	1×10^{6} pM
S20	Fabry-Pérot cavity enhanced SPR	angular spectrum of absorption	gold coated Fabry-Pérot cavity	COVID antigen MBP-CoV-2NP (N/A)	100 pM

Reference

S1 Wang, Y. *et al.* Targeted sub-attomole cancer biomarker detection based on phase singularity 2D nanomaterial-enhanced plasmonic biosensor. *Nano-micro letters* **13**, 1-11 (2021).

S2 Němec, P. *et al.* Ge–Sb–Te thin films deposited by pulsed laser: An ellipsometry and Raman scattering spectroscopy study. *Journal of Applied Physics* **106** (2009).

S3 Park, J.-W. *et al.* Optical properties of pseudobinary GeTe, $Ge_2Sb_2Te_5$, $GeSb_2Te_4$, $GeSb_4Te_7$, and Sb_2Te_3 from ellipsometry and density functional theory. *Physical Review B* **80**, 115209 (2009).

S4 Palik, E. D. *Handbook of optical constants of solids*. Vol. 3 (Academic press, 1998).

S5 Jiang, Y., Pillai, S. & Green, M. A. Realistic silver optical constants for plasmonics. *Scientific reports* **6**, 30605

(2016).
S6 Mitchell, J. S. & Lowe, T. E. Ultrasensitive detection of testosterone using conjugate linker technology in a nanoparticle-enhanced surface plasmon resonance biosensor. *Biosensors and Bioelectronics* **24**, 2177-2183 (2009).
S7 Mitchell, J. Small molecule immunosensing using surface plasmon resonance. *Sensors* **10**, 7323-7346 (2010).
S8 Yuan, J. *et al.* Sensitivity enhancement of SPR assay of progesterone based on mixed self-assembled monolayers using nanogold particles. *Biosensors and Bioelectronics* **23**, 144-148 (2007).
S9 Riskin, M. *et al.* Molecularly imprinted Au nanoparticles composites on Au surfaces for the surface plasmon resonance detection of pentaerythritol tetranitrate, nitroglycerin, and ethylene glycol dinitrate. *Analytical Chemistry* **83**, 3082-3088 (2011).
S10 Frasconi, M. *et al.* Surface plasmon resonance analysis of antibiotics using imprinted boronic acid-functionalized Au nanoparticle composites. *Analytical Chemistry* **82**, 2512-2519 (2010).
S11 Gnedenko, O. V. *et al.* Highly sensitive detection of human cardiac myoglobin using a reverse sandwich immunoassay with a gold nanoparticle-enhanced surface plasmon resonance biosensor. *Analytica chimica acta* **759**, 105-109 (2013).
S12 Bai, Y. *et al.* Aptamer/thrombin/aptamer-AuNPs sandwich enhanced surface plasmon resonance sensor for the detection of subnanomolar thrombin. *Biosensors and Bioelectronics* **47**, 265-270 (2013).
S13 Liang, R.-P. *et al.* Magnetic Fe3O4@ Au composite-enhanced surface plasmon resonance for ultrasensitive detection of magnetic nanoparticle-enriched α-fetoprotein. *Analytica chimica acta* **737**, 22-28 (2012).
S14 Sreekanth, K. V. *et al.* Extreme sensitivity biosensing platform based on hyperbolic metamaterials. *Nature materials* **15**, 621-627 (2016).
S15 Minopoli, A. *et al.* Ultrasensitive antibody-aptamer plasmonic biosensor for malaria biomarker detection in whole blood. *Nature communications* **11**, 6134 (2020).
S16 Cetin, A. E. *et al.* Handheld high-throughput plasmonic biosensor using computational on-chip imaging. *Light: Science & Applications* **3**, e122-e122 (2014).
S17 Yesilkoy, F. *et al.* Phase-sensitive plasmonic biosensor using a portable and large field-of-view interferometric microarray imager. *Light: Science & Applications* **7**, 17152-17152 (2018).
S18 Liu, L. *et al.* Ultrasensitive detection of endocrine disruptors via superfine plasmonic spectral combs. *Light: Science & Applications* **10**, 181 (2021).
S19 Kabashin, A. V. *et al.* Plasmonic nanorod metamaterials for biosensing. *Nature materials* **8**, 867-871 (2009).
S20 Allison, G. *et al.* A Fabry-Pérot cavity coupled surface plasmon photodiode for electrical biomolecular sensing. *Nature Communications* **12**, 6483 (2021).
